# Psychophysiological Regulation and Classroom Climate Influence First and Second Graders’ Well-Being: The Role of Body Mass Index

**DOI:** 10.3390/ejihpe11040112

**Published:** 2021-12-03

**Authors:** Sara Scrimin, Marta Peruzza, Libera Ylenia Mastromatteo, Elisabetta Patron

**Affiliations:** 1 Department of Developmental Psychology and Socialization, University of Padova, Via Venezia, 8, 35151 Padova, Italy; marta.peruzza@gmail.com (M.P.); liberaylenia.mastromatteo@phd.unipd.it (L.Y.M.); 2 Department of General Psychology, University of Padova, Via Venezia, 8, 35151 Padova, Italy; elisabetta.patron@unipd.it

**Keywords:** physiological regulation, classroom climate, body mass index, well-being, heart rate variability, vagal withdrawal

## Abstract

This study examines the associations between physical and emotional well-being and classroom climate, cardiac vagal response, and body mass index (BMI) in a sample of 6- to-8-year-olds. Specifically, we expected a direct link between classroom climate, vagal withdrawal, BMI and children’s physical and emotional comfort. Furthermore, we explored whether these individual and environmental characteristics influenced well-being in an interactive fashion. Participants were 142 (63 boys, 44%) first and second graders living in the North of Italy who were interviewed on their emotional and physical comfort. Heart rate and a measure of vagal influence on the heart (cardiac vagal tone) were recorded at rest and during an oral academic test. Height and weight were collected. Classroom climate was positively linked with physical well-being, whereas emotional well-being was negatively related with BMI. In addition, an inverted U-shaped effect of cardiac vagal withdrawal (i.e., cardiac vagal tone during stress minus resting vagal tone) on emotional well-being was found. Two regression models highlighted the role played by BMI when interacting with vagal withdrawal in predicting children’s physical and emotional well-being. The interplay between BMI and cardiac vagal withdrawal played an important role in primary school children’s well-being. From a clinical perspective, preventive training to improve autonomic regulation in concert with interventions promoting healthy eating attitudes might be critical for supporting primary school children’s emotional and physical health.

## 1. Introduction

The first years of primary school represent an important period in children’s lives during which they enter a new environment characterized by a number of new social and academic challenges [1]. School provides a context rich in positive stimuli but also dotted with possible stressors; if and how students are capable of dealing with these challenges influences their well-being [2,3]. For example, students have to deal with cognitive requests, the pressure of being evaluated and the difficulties related to constant social interactions within a new environment and, often, with new mates. The ability to cope with such stressors increases well-being and chances for future success [2,4].

Great emphasis is placed on child well-being [4,5] indexed by general physical health perception and emotional comfort [6]. While child well-being is a complex and multifaceted concept [7], scholars agree on the importance of biological functioning, somatic symptoms, and functional status as indexed by general physical health perception. To complete the picture, emotional comfort as indexed by the frequency of emotional difficulties related to, e.g., fear, discomfort, sadness or rage must be addressed [6,8].

Importantly, it has been shown that environmental and individual factors, together with their continuous interaction, determine the child’s well-being both in the short-and long-term [4]. How students adapt to the school environment and deal with the repetitive challenges that they encounter is influenced by both individual and contextual variables [9], including individual differences in self-regulation [3] and stress response [10] as well as their perception of the classroom climate [11].

Furthermore, all aspects of child development, including socioemotional well-being, are dependent upon nutrition and correlated with overweightness and thinness [12]. Researchers and policymakers are increasingly recognizing the interdependence of several aspects of health and well-being. However, little is known about the role played in well-being by the interplay between a more or less healthy body mass index (BMI), the child’s everyday environment and self-regulation.

Transactions between individual, environmental and biological factors are constantly occurring [4]. Following a biopsychosocial model, we examined whether first and second graders’ well-being is directly or interactively linked with their cardiac vagal response to an oral academic test, perceived classroom climate and body weight.

### 1.1. Cardiac Vagal Response to Academic Test

Academic tests are usually perceived by students as challenging stressful situations that elicit a specific cardiac response [13]. The activity of the heart is strictly regulated by the two branches (sympathetic and parasympathetic) of the autonomic nervous system (ANS) [14]; on the one hand, sympathetic activation determines increases in heart rate (HR) and stroke volume, and on the other hand, parasympathetic activation of the heart is achieved through the vagus nerve, which acts as a “brake” on HR [15]. When the vagus nerve is active, HR slows down; when the “brake” is removed, the heart beats faster. The ability to inhibit vagal tone during a challenging stressful situation has been identified as an important index of the adaptive response [16,17]. A moderate cardiac vagal withdrawal (leading to a moderate HR increase) during challenging stressful situations has been linked to better performance and emotional well-being in the context of both emotional and cognitive environmental challenges [18,19,20]. Research found that during a stressful task, both excessive [21] and blunted [18,19] vagal withdrawal predicted poorer outcomes in children. Specifically, excessive vagal withdrawal (i.e., excessive HR increase) was found to correlate with greater manifestation of negative affect (sadness and anger) in a social challenge task [21], and to characterize children at risk for behavior problems [16]. Blunted vagal withdrawal (i.e., HR reduction) was found to correlate with coping difficulties and externalizing behaviors [16,17], and with poor performance in executive function tasks in children [18,19]. Thus, it could be argued that adequate (i.e., moderate) vagal withdrawal during a challenging stressful task is a reliable predictor of children’s well-being, reflecting children’s emotional and cognitive self-regulation abilities [17].

### 1.2. Classroom Climate

In addition to individual variability in terms of regulation when facing school challenges, the way students feel in relation to their classroom is also of paramount importance. The way children perceive and respond to stimuli is strongly related to the environment they experience now and have experienced in the past [17,22]. Specifically, unsafe, threatening or challenging contexts may repetitively trigger a stress response in students and require the dissipation of a great amount of energy to regulate it [23]. In addition, past experiences related to a specific environment might generate negative or worrisome expectations that cause or reinforce a stress response that needs to be downregulated in order to function adequately in terms of emotional and physical well-being [24]. Classroom emotional climate has been defined as the extent to which teachers and students form a harmonious microsystem with high quality social and emotional interactions both between and among students and teachers [25]. Literature confirms the existence of a close connection between students’ perception of the classroom climate and their psycho-social [26] and academic outcomes [27]. Empirical evidence suggests that children’s perception of a positive classroom climate is linked to emotional well-being [26] and less physical distress [27]. Research on school climate has focused on the role of caring school communities emphasizing the importance of the social and interpersonal aspects of the school experience and their relevance to students’ well-being [28]. Most importantly, longitudinal findings indicate that caring school environments foster cooperation, spontaneous prosocial behavior, and supportive ties among students, which are related to better scholastic performance and greater long-term well-being [29,30].

It should be noted that studies on classroom climate usually measure this variable with methods ranging from direct observations of classroom environments to interviews with teachers [31], yet as valuable as these methods are for providing insights into classroom context, pupils’ own perceptions of their classroom environment may offer more valuable information [32]. Classroom climate is usually considered a group-level construct that is experienced equally by all students in the class; however, considering personal evaluations might highlight students’ individual experiences in the class: students may have different relationships with their teacher, different abilities to deal with challenges and might perceive the same environment differently. Given that a large part of children’s days is spent at school, understanding the effects of classroom climate on well-being also in relation to specific students’ individual characteristics (such as their emotional regulation in response to stress) may provide important insights which can be useful for developing school-based prevention and/or intervention programs.

### 1.3. Body Mass Index

Child well-being is closely interconnected with physical and nutritional health [33]. A good indicator of child nutrition, as an estimate of total body fat in children and adolescence, is body mass index (BMI)—calculated as weight (kg)/height squared (m^2^) [34]. A healthy BMI is associated with better development and less impeded growth throughout infancy and childhood [12]. In recent years, with the increase of obesity rates in childhood, several studies have been conducted assessing the relationship between overweightness and health related issues [35] as well as lack of self-esteem [36], presence of mental health problems [37] and poor academic achievement [36]. These studies suggest that body weight is negatively correlated with emotional and physical well-being.

However, although obesity and mental health problems have been often reported as comorbid conditions, it should be noted that this seems to be a bidirectional association. Hunsberger and colleagues [38] conducted a longitudinal study finding that children who were overweight at baseline had increased risk of poor health-related quality of life two years later; but it was also found that poor well-being at baseline was associated with increased risk of being overweight at follow-up. Interestingly, previous works have also demonstrated the existence of a link between stress and BMI. For example, high hair cortisol concentration (HCC), an index of activation of a stress response, was associated with higher BMI in a large sample of six-year-old children [39] and obese children had higher HCCs than non-obese peers [40]. In addition, Gerber and colleagues [41] found significant relationships between HCCs, BMI and somatic complaints. However, these results are in contrast with another study that considered children living in disadvantaged neighborhoods [42]. Such variety in findings on the link between BMI and well-being might be explained by the existence of different moderating factors, such as children’s individual characteristics and the child’s environment, that could be relevant for explaining the link between BMI and well-being.

### 1.4. Cardiac Vagal Tone and BMI

Cardiac vagal response to stress is a fairly good index of how individuals respond and adapt to challenges [15]. This physiological self-regulatory ability is linked to well-being but can also be strictly interconnected with food intake, and more specifically with energy consumption and consequently BMI. It has been documented that exposure to stressful environments (defined as contexts characterized by an ongoing or anticipated threat to well-being, and lack of a harmonious microsystem with poor social and emotional interactions) evokes a constellation of physiological and behavioral responses that markedly alter metabolic and behavioral states in humans and animals [43]. The ability to regulate such physiological responses might, as a consequence, help to promote adequate food intake and functional energy consumption. Longitudinal studies found that high levels of emotional reactivity, irritability and impulsivity, and low levels of persistence and self-regulation, were related to higher BMI in children [20,44,45,46,47]. In this context, the ability of the child to self-regulate in response to a challenging stressful situation, such as an academic test, might play a relevant role in interaction with BMI.

### 1.5. Classroom Climate, BMI and Well-Being

It has been shown that spending time in negative emotional contexts can alter both the quantity and quality of calories consumed, and stress-induced alterations in food intake can also interact with individuals’ emotional states [48]. However, the relationship between environment and food intake/BMI is ambiguous. Negative and stressful environments have been shown to both increase and decrease caloric intake, and numerous reports indicate that chronic stress exposure can promote either obesity or anorexia within certain dietary environments.

It has been shown that quality of living environment is linked with BMI. For example, Cohen and colleagues [49] found that the quality of their neighborhood was significantly related to adolescents’ BMI. Similarly, the classroom climate that children experience at school can be related to BMI. However, more data are needed to understand whether the relationship is positive or negative, particularly among school-age children. Stress exposure can influence eating behaviors in children as young as eight years of age [50]; hence, we could expect exposure to negatively perceived stressful classroom environments to be linked to dysfunctional food intake.

It has been documented that exposure to different sources of distress often precedes the consumption of high calorie, low nutrient foods [50,51,52], possibly causing weight gain and increase in BMI. However, other studies, in older children, showed a decrease in food intake, also related to accelerated energy consumption, when exposed to negatively perceived contexts [53]. These findings are supported by the notion that suppression of feeding is adaptive and promotes survival when fear is triggered by real danger [53], yet of course can become maladaptive when sustained fear halts feeding under energy-depleted states, such as in anorexia nervosa [54].

Spending time in environments characterized by a negative emotional climate might have a significant impact on food intake and body weight. However, data on children are scarce. The majority of existing studies focus mainly on the link between a pathological weight status (above or below average) and individuals’ psychological and physical functioning; yet it might be useful to consider whether even variations within the normal range of children’s BMI are associated with well-being. Specifically, it might be that at a young age, normal variation in body weight could interact with individual or environmental characteristics in determining well-being. It might be that even small variations in BMI could be possible risk factors for the development of subsequent weight problems and mental health disorders.

Understanding the effects of within normal BMI variations might help to identify possible risk factors in relation to weight before they become pathological. Given the facility of monitoring children’s BMI and the objectivity of the data, this could be valuable information for early detection of more at-risk students. This could possibly promote targeted interventions to prevent the development of severe distress.

### 1.6. The Present Study

The present cross-sectional study aims to expand the reported literature by simultaneously examining the influence of classroom climate, ability to self-regulate in a challenging stressful situation (vagal withdrawal) and BMI on children’s physical and emotional well-being. The rationale is that of better understanding the role of environmental and individual factors in affecting children’s well-being at the beginning of primary school. In addition, a special focus is placed on BMI as a potential objective factor that might buffer this relationship among children with a normal body weight. Specifically, two research questions guided the study: do classroom climate, vagal withdrawal and BMI influence first and second graders’ (RQ1) physical comfort and (RQ2) emotional comfort in a (a) direct or (b) interactive fashion while controlling for age and gender?

In terms of direct effects, satisfaction with classroom climate was expected to be directly and positively associated with both children’s physical and emotional comfort. Previous literature has reported that children spending time in a positive classroom environment characterized by supportive relationships with their teachers and peers is linked to greater well-being, both in the short and in the long term [25]. Hence, we expected children that reported a positive classroom climate to also experience more emotional comfort and fewer somatic complaints. Furthermore, based on previous literature, vagal withdrawal was expected to be negatively associated with both children’s physical and emotional comfort [18,19,20]. Last, BMI was expected to be directly and negatively related to students’ well-being. Furthermore, in terms of indirect effects, we expected to find that the three independent variables (i.e., classroom climate, vagal withdrawal and BMI) interacted in predicting students’ well-being. However, given the scant literature assessing such relationships, it was difficult to advance precise hypotheses. To advance some hypothesis in relation to the two-way interaction, we might expect the existence of an interaction between vagal withdrawal and classroom climate, with both average vagal withdrawal and better classroom climate working as protective factors in promoting physical and emotional comfort [15,25]. Similarly, we could hypothesize cardiac vagal activity to interact with BMI, where the ability to regulate in the face of a challenge could be linked with a satisfactory BMI and greater perceived well-being [44]. Lastly, a more positive classroom climate could be linked with an average BMI and physical/emotional comfort, whereas a negative environment might be associated with either under- or overweight and less satisfied children [54]. The three-way interaction would also be tested. Our expectation here would be to have a positive classroom climate and moderate vagal withdrawal linked with healthy BMI and consequently greater well-being. Hence, both environmental and individual factors could work as protective factors in promoting a healthy weight and satisfying emotional and physical comfort.

## 2. Materials and Methods

### 2.1. Participants

We enrolled 142 first and second graders (63 boys, 44%) in this study with a mean age of 6.82 years (SD = 0.71). Students attended five (a total of seven classes were involved in the study) public primary schools in Northern Italy. Schools were selected from participant institutions in a larger intervention project aimed at reducing educational poverty. All families came from lower or middle class families. Specifically, 60% of families involved in this study had a yearly income of less than 12,000 EUR, which is consistent with low SES in Italy according to the National Institute of Statistics (ISTAT, 2020); the remaining families had an income between 12,000 and 30,000 EUR. Furthermore, 75% of families had just one working parent. Children with cognitive or psychological disorders, cardiovascular disorders, or other medical conditions were excluded from the study.

### 2.2. Procedure

The study was approved by the Research Ethics Committee of the University of Padova (protocol: 2111, approval number: 89ADC65ECC5E40203FF0079D9D6CDB53); in addition, written informed consent was obtained from school principals. For participation, we required both parental written permission and students’ verbal consent. Data were collected between January and February 2019. Before the beginning of data collection and recording, trained researchers spent three months participating in classroom activities and organizing games with children in order to familiarize with students and obtain students’ total trust (the familiarization period occurred between October and December 2018). Children were subsequently tested individually in a quiet room at school. All recordings took place in the morning (between 9 a.m. and 12 p.m.). After attachment of sensors, the researcher invited the child to sit comfortably and watch an eight-minute-long relaxing film-clip (“Spot’s Lost Bone”; King Rollo Films, 1987) during which an electrocardiogram (ECG) was recorded. After the film-clip was over, and in order to create a situation of mild stress reproducing that of an oral test in the classroom after a lesson, an interview including 15 questions (lasting 3 min) was conducted. At the end of the interview the sensors were removed, and the child was repeatedly praised on his/her performance. After this the child was asked to report on (a) the difficulty he/she had experienced while answering the questions, (b) satisfaction with classroom, peers and teachers, and (c) perception of his/her own health and well-being in terms of physical and emotional comfort. Weight and height were assessed (without shoes) at this time in order to avoid a post-prandial state. Last, the child was debriefed before returning to class.

It is important to note that all the children were familiar with the researcher who collected the data and were happy to join her for the individual session.

### 2.3. Measures

#### 2.3.1. Child’s Perception of His/Her Health and Well-Being

The Child Health and Illness Profile—Child Edition (CHIP–CE) [55] is a questionnaire composed of 45 items that can be administered to a child as an interview. It evaluates the well-being of 6–11-year-old children and examines several aspects of health and well-being that can be influenced by health systems, school systems, and health promotion efforts. The CHIP–CE allows the collection of information on health-related quality-of-life aspects that are of special interest to school-aged children. In the present study, we used the Emotional and Physical Comfort items (i.e., experience of emotional and physical symptoms and observed activity limitations). Frequency of symptoms in the past four weeks was assessed using a five-point Likert scale. The measure is well-known for its great psychometric properties [55]. In the present study, both subscales had good internal consistency (Cronbach Alphas 0.76 and 0.80, respectively).

#### 2.3.2. Child’s Perception of Classroom Climate

A standardized set of five questions adapted from the social support scale of the CHIP–CE [56] were asked regarding satisfaction with social relationships occurring within the classroom with peers (e.g., “How satisfied are you with the classmates you have?”), teachers (e.g., “How satisfied are you with your math teacher?”), physical environment (e.g., “How satisfied are you with what your classroom looks like?”) and emotional environment (e.g., “How satisfied are you with the emotional support and help you receive while spending time in your classroom?”). Questions assessed the degree of satisfaction using a five-point Likert scale (from “not at all satisfied/happy” to “very satisfied/happy”). The measure has been previously used in this exact form and has shown excellent psychometric properties [3]. In terms of internal consistency, in the present study Cronbach’s Alpha coefficient for the scale was 0.81.

#### 2.3.3. Simulation of a School Oral Test

The child was questioned on the film-clip story he/she had just seen. Two researchers told the child: “Now we will question you on what you have just seen. This is a listening comprehension test, you must answer as best and as precisely as possible to all the questions.” A standard set of 15 questions was asked. Specifically, 10 questions were focused on single events and general characteristics of the animated story (i.e., “Where did Spot look for his bone at first?”), while five questions were focused on more specific events or multiple details of the video-clip (i.e., “How many dots does Spot have on his back?”). In order to maintain a uniform level of child stress during the test and to make it similar to a school test, instructions were given between each question (i.e., “Ok, but be careful because the next question is going to be hard!”, “Be careful, try to remember as many details as possible!”). All children answered all the questions.

#### 2.3.4. Psychophysiological Measure

To record the ECG signal, a POLAR sensor was positioned on the child’s thorax using a physiological monitoring device that encodes biological signals (ProComp Infiniti, Thought Technology, Montreal, QC, Canada). The ECG signal was recorded via a 12-bit analog-to-digital converter with a sample rate of 256 Hz and stored sequentially for analysis. Then, the raw ECG signal was exported into Kubios-HRV 2.2 (Kuopio, Finland), which is software able to estimate the occurrence of each heartbeat and derive the series of inter-beat intervals (IBIs; computed as the difference in ms between successive R-waves). To identify and correct artifacts, we visually inspected the raw signal, and a piecewise cubic splines interpolation method was performed. Then, we calculated the square root of the mean squared differences (rMSSD) of successive IBIs. rMSSD is an index of short-term heart period fluctuations and reflects vagally mediated influence of the parasympathetic activity on the sinoatrial node [56]. To compute rMSSD change, we subtracted rMSSD at baseline from rMSSD during the stress task.

#### 2.3.5. Body Mass Index

Weight was measured using an electronic scale to the nearest 0.1 kg in light clothing and without shoes. Height was measured to the nearest 0.1 cm using a stadiometer. BMI was calculated as weight in kilograms divided by the square of height in meters [57,58]. The BMI score was transformed into standardized BMI z-scores that were adjusted for age and gender using data obtained from the World Health Organization reference dataset for children between five and 19 years [59].

### 2.4. Statistical Methods

Analyses were performed using R software (R Core Team, 2016). All participants with one or more missing data points were deleted from the data set. Missing data accounted for 0.2% of the total sample and were due to HRV measurement problems which did not allow accurate HR recording. Prior to analysis, data were checked for univariate and multivariate normality. Univariate normality was determined for each variable through examination of skewness and kurtosis. Multivariate normality was assessed using Mardia’s Coefficient [60], which evaluates multivariate normality through evaluation of multivariate kurtosis. Group means and Pearson’s correlation between all study variables were obtained as preliminary analyses. In addition, we explored the potential quadratic relationship between the two dependent variables and both BMI z-scores and rMSSD changes. Subsequently, at the multivariate level, two series of linear regression models were conducted, one for physical and one for emotional well-being. Starting from the baseline theoretical model, we used a model selection approach based on Akaike Information Criterion (AIC) to find the most plausible model based on the observed data (AIC) [61,62]. In all tested models, we included all main effects of our study variables (i.e., child’s perception of classroom climate, rMSSD change and BMI z-scores) as well as their two- and three-way interactions. In addition, age and gender were included as control variables in the models. We relied on an exploratory rather than confirmatory model selection approach, based on the assumption that children’ physical and emotional well-being are very complex phenomena which can hardly be captured in a single confirmatory model. Results were interpreted by calculating an evidence ratio comparing the AIC of the best fitting model to the AIC of the baseline model [61,62]. To test interaction effects, simple slope tests were performed and then, to identify the regions of significance for each moderated effect, Johnson–Neyman intervals were calculated [63] using the package jtools [64] in R. In addition, significant interactions were plotted at the mean level and at one standard deviation (SD) above and below the mean of the moderator. A graphical representation of the regions of significance for each interaction is provided.

## 3. Results

### 3.1. Preliminary Analyses

Data distribution was proven normal, both in terms of graphical representation and skewedness and kurtosis testing. Moreover, in accordance with most social and behavioral science data [65], examination of the Mardia’s coefficient suggested multivariate normality of data. In preliminary analyses, we computed group means and bivariate correlations (see Table 1). BMI row scores were also normally distributed (*M* = 17.08, *SD* = 3.03, range = 12.10–30.72) and overall, in our sample, 29 children (21%) were overweight (BMI above 19-Z-score > 1.04) and nine (6%) were obese with a Z-score > 1.64 (see Supplementary Figure S1 for frequency distribution). As can be seen in Table 1, children’s physical and emotional well-being were positively correlated. In addition, children’s perception of classroom climate was not so strongly but still positively linked with physical well-being, whereas emotional well-being was more significantly and negatively related to BMI z-scores. In addition, a significant quadratic relationship was found between emotional well-being and rMSSD change, *F*(2,141) = 4.65, *p* = 0.02, resulting in an inverted U-shaped effect of HRV changes on emotional well-being. All other tested quartic relations were non-significant.

### 3.2. Association between Classroom Climate, Body Weight, Psychophysiological Regulation and Physical Comfort (RQ1)

**Figure 1:**
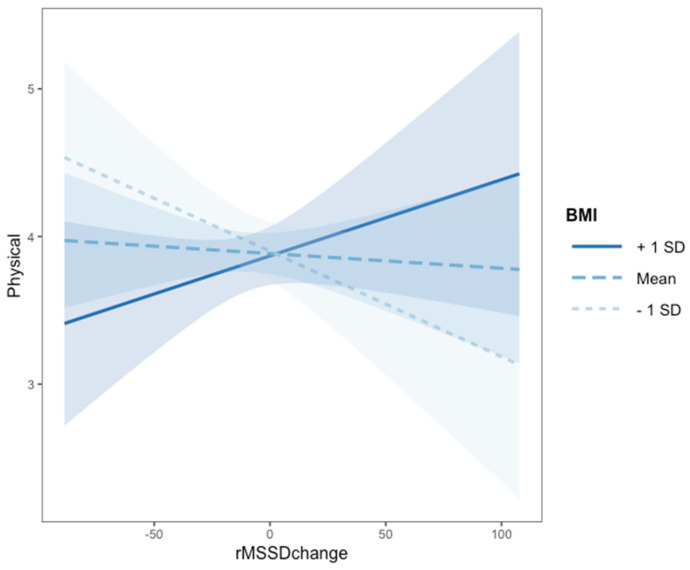
Interaction effect of rMSSD change and BMI on physical comfort (N = 130). *Note.* The Johnson–Neyman technique identified that rMSSD change had a significant effect on physical well-being only when zBMI was lower than −2.51, that is, when children were in the underweight range, and higher than 3.82 (not applicable to our sample), that is, when children were in the obese range.

To test the first research question (RQ1) on children’s physical comfort we included in the model the main effects of children’s perception of classroom climate, rMSSD change and BMI z-scores, as well as their two-and three-way interactions, while controlling for age and gender. The best fitting model (AIC = −63.6; A ICw = 0.50) predicting physical comfort is presented in Table 2, Panel A. The associated evidence ratio showed that this model was 69 times more likely to have generated the observed data than the baseline model (AIC = −55.1). This model explained 14% of the variance in physical well-being. The model included a significant main effect of rMSSD change on children’s perception of classroom climate. Moreover, an interaction between rMSSD change and BMI z-scores (zBMI) was found (Figure 1). To explore the interaction effect, we performed tests of the simple slopes (Aiken & West, 1991). As can be seen in Figure 1, children with low zBMI and reduced rMSSD change also reported lower physical well-being (*B* = −0.05, *SE* = 0.03, *t* = −1.87, *p* = 0.04), whereas children with normal to high zBMI (*B* = 0.02, *SE* = 0.04, *t* = 1.29, *p* = 0.21) seemed to be less affected by their psychophysiological response to stress (i.e., rMSSD change).

In fact, analyses of the interaction using the Johnson–Neyman technique identified that rMSSD change had a significant effect on physical well-being only when zBMI was lower than −2.51, that is, when children were in the underweight range, and higher than 3.82 (not applicable to our sample), that is, when children were in the obese range (see Supplementary Figure S2).

### 3.3. Association between Classroom Satisfaction, Psychophysiological Regulation, Body Weight and Emotional Comfort (RQ2)

**Figure 2:**
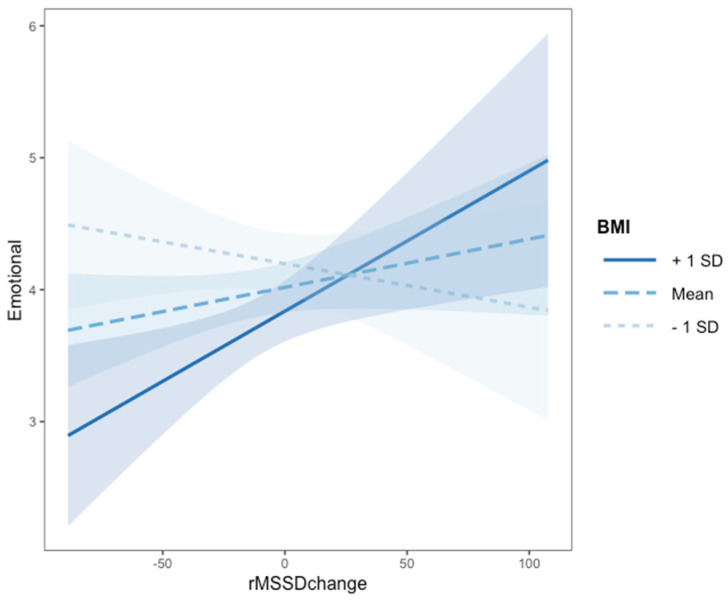

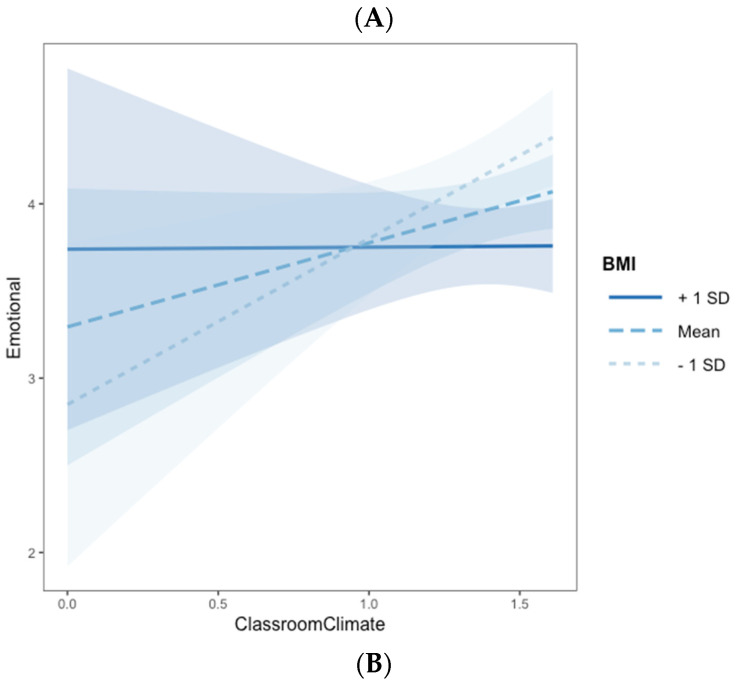
Interaction effects of rMSSD change with BMI Panel (**A**) and of satisfaction with classroom climate with BMI Panel (**B**) on emotional comfort (N = 130). *Note.* Panel (**A**). The Johnson–Neyman technique identified that rMSSD change had a significant effect on emotional well-being for values of zBMI lower than −3.65 (not applicable to our sample) and higher than −2.15, hence being within a normal range. Panel (**B**). The Johnson–Neyman technique identified that satisfaction with classroom climate had a significant effect on emotional well-being for values of zBMI lower than −2.57, that is, between thinness and underweightness, and higher than 5.84 (the latter is not applicable to our sample).

To test the second research question (RQ2) regarding children’s emotional comfort we included in the model the main effects of children’s perception of classroom climate, rMSSD change and BMI z-scores, as well as their two- and three-way interactions, while controlling for age and gender. The best fitting model predicting emotional well-being (AIC = −87.6; AICw = 0.54) is presented in Table 2, Panel B. The associated evidence ratio showed that this model was 86 times more likely to have generated the observed data than the baseline model (AIC = −84.65). This model explained 15% of the variance in emotional well-being. Results indicated that satisfaction with children’s perception of classroom climate was directly associated with emotional well-being as well as rMSSD change. Furthermore, two interactions emerged: zBMI interacted with both rMSSD change and satisfaction with classroom climate in predicting emotional well-being. To explore the two-way interaction effect, we performed tests of the simple slopes (Aiken & West, 1991). As can be seen in Figure 2 (Panel A), children with high zBMI showing greater rMSSD change reported lower emotional well-being (*B* = 0.01, *SE* = 0.04, *t* = 2.61, *p* = 0.01). Children with low zBMI (*B* = −0.01, *SE* = 0.01, *t* = −0.92, *p* = 0.36) seemed to be less affected by their psychophysiological response to stress (i.e., rMSSD change). Analyses of this interaction using the Johnson–Neyman technique identified that rMSSD change had a significant effect on emotional well-being for values of zBMI lower than −3.65 (not applicable to our sample) and higher than −2.15, hence being within a normal range (see Supplementary Figure S3 Panel A).

In relation to the interaction between children’s perception of classroom climate and zBMI, as displayed in Figure 2 (Panel B), children with low zBMI were significantly affected by their perception of classroom climate and reported higher emotional well-being when they were more satisfied with their classroom (*B* = 0.95, *SE* = 0.34, *t* = 2.84, *p* = 0.005). Emotional well-being of children with higher zBMI did not change as a function of their perceived classroom climate (*B* = 0.02, *SE* = 0.37, *t* = 0.13, *p* = 0.97). Analyses of this interaction using the Johnson–Neyman technique identified that satisfaction with classroom climate had a significant effect on emotional well-being for values of zBMI lower than −2.57, that is, between thinness and underweightness, and higher than 5.84 (the latter is not applicable to our sample) (see Supplementary Figure S3 Panel B).

## 4. Discussion

The present study addressed the link between physical and emotional well-being and children’s perception of classroom climate, cardiac vagal responses to an oral academic test and BMI z-scores among six- to-eight-year-olds. The main findings highlighted a role played by BMI z-scores interacting with vagal withdrawal in predicting children’s emotional and physical well-being. Moreover, BMI z-scores also interacted with the children’s perception of classroom climate in determining their emotional well-being.

As expected, satisfaction with classroom climate was linked with greater emotional and physical well-being, as reported by children during the interview. Although the relationship was weak, this is in line with a great amount of literature underlining the importance of a positive perception of classroom climate for students’ well-being [27]. Previous studies have shown that when the classroom is perceived as safe and good relationships are experienced during classroom hours, students not only improve their grades and academic performance [66,67] but also report less physical distress and greater emotional well-being.

No direct association was found between vagal withdrawal and children’s well-being. However, children’s emotional well-being and cardiac vagal withdrawal were quadratically associated in a U-inverted way. This is in line with previous literature reporting that moderate cardiac vagal withdrawal predicts children’s well-being and performance in challenging situations [18,19]. By contrast, both excessive [21] and blunted [18,19] vagal withdrawal have been found to predict poor outcomes in children. These data suggest that excessive cardiac vagal withdrawal to challenging stressful situations might mark one or more core self-regulatory functions being disrupted across diverse forms of psychopathology [68], whereas moderate vagal withdrawal support better self-regulation abilities and overall functioning.

BMI was found to be negatively and directly linked with children’s perceived emotional well-being. Despite the smallness of the effect, and the fact that within our sample just 6% of children were considered obese, this supports the existing literature reporting a higher number of behavioral and emotional problems in obese children compared with normal weight ones [69], including poorer self-esteem [36] and higher display of depressive symptomatology [37] in children with a clinically high BMI. The body weight of children taking part in the study was not clinically over-or under-weight but normally distributed, with the majority being within the healthy weight range [58,70]. Nevertheless, the present results suggest that there is a tendency for children with higher BMI to be more vulnerable to emotional difficulties compared to thinner ones. This finding should be further explored in order to try to understand whether it is the insufficient emotional well-being that causes an increase in BMI, or if it is the BMI itself or even the negative social stigma of having a high BMI that causes poor emotional comfort. The present data might also be a further confirmation of the significant amount of literature linking physical activity with well-being [71]; that is, children that have a greater BMI and lower well-being might also perform less physical activity. Considering the young age of our sample, just 20% of the participants could be considered overweight, but body weight might increase later on (even reaching obese levels), especially if in the meanwhile they did not benefit as much as others from regular physical activity.

In order to study the possible interacting roles of classroom satisfaction, rMSSD change and BMI in predicting children’s emotional and physical well-being while controlling for age and gender, two best fitting models were identified. BMI was found to mildly moderate the relationship between rMSSD changes during a challenging stressful task, satisfaction with classroom climate and children’s physical and emotional well-being. Regarding children’s physical well-being, analyses revealed that only among children with a low BMI z-score (<−2.5), that is, under-weight (BMI below the 15th percentile) [72], was greater cardiac vagal withdrawal associated with better physical well-being and lower vagal withdrawal linked with poorer physical well-being. The opposite trend was found among healthy weight children, but it was non-significant. These data might indicate that being able to self-regulate more when facing daily challenges is particularly important for thinner children’s physical comfort. This could be related to the fact that children with high energy consumption and/or lower calorie intake (lower BMI), when unable to regulate, might perceive their physical distress more.

First and second graders’ emotional well-being was predicted by the interaction between BMI and rMSSD changes during a challenging stressful task, as well as by the interaction between BMI and classroom satisfaction. Taken together, these data support a biopsychosocial model and underline the importance of the continuous transactions between children’s individual self-regulatory abilities, classroom climate and BMI. In relation to the first interaction, slope analyses revealed that only among children with a healthy weight (between the 15th and 75th percentile) [70] was a higher cardiac vagal withdrawal associated with worse emotional well-being and a moderate vagal withdrawal linked with greater emotional well-being. The opposite trend was once again found among children with lower BMI, but it was non-significant. Cardiac vagal withdrawal during a challenging stressful situation seems to differently predict physical and emotional well-being in children with lower compared to higher BMI. Results showed that among children with lower BMI, those who responded to the test with greater vagal withdrawal reported better physical and emotional well-being compared to the ones with no vagal changes in terms of withdrawal or augmentation. These data were significant only for physical well-being, but the trend was very similar for emotional well-being. Among children with a healthy weight, findings revealed an opposite slope where only those who responded with moderate or no vagal withdrawal to the test showed better physical and emotional well-being. Here the slope was significant only for emotional well-being, but the trend was very similar for children’s physical health. Previous study linked excessive [21] and blunted vagal withdrawal [19] to poorer emotional and cognitive outcomes in children. Similarly, excessively high BMI has also been linked to worse emotional outcomes, and low BMI has been shown to be associated with psychopathology, such as anxiety symptoms [20]. Importantly, underweight children have been shown to suffer from internalizing and externalizing problems compared to children with normal weight [33]. It could be hypothesized that children with low, compared to high, BMI may use different strategies to face challenging stressful situations. Our data suggest that children with lower BMI benefited from higher cardiovascular response, while by comparison, children with higher BMI and moderate cardiac vagal withdrawal reported better well-being. To our knowledge this is the first study to investigate the interaction between BMI and cardiac vagal response to a challenging situation in school. From the present findings it could be argued that, taken together, BMI and vagal withdrawal are more informative of a child’s well-being compared to the single factors. Consequently, it might be worth considering both these factors, particularly when evaluating children’s responses to stress and its effect on well-being.

In relation to the second interaction, slope analyses revealed that BMI moderated the link between classroom satisfaction and emotional well-being only among children with low BMI z-scores. That is, children reporting worse emotional well-being had lower BMI and were less satisfied with the classroom climate as they perceived it. This result could be interpreted in light of previous findings linking exposure to negative and stressful environments with low food intake end high energy consumption [53,54]. Even though the present data are not longitudinal and we cannot infer causality, we might propose that children that on a daily basis experience a negative classroom climate, where they do not experience positive relationships and are not valued but instead criticized, respond by activating a fear response. Such a response, in the context of real danger, takes priority over feeding, which is less important than safety in risky environments. This might explain the relationship between low BMI and poor emotional well-being in negatively perceived classroom contexts. By comparison, children with higher BMI z-scores reported a medium-high emotional well-being independently of classroom climate satisfaction. It has to be noted that lower BMI has been consistently associated with a greater sensitivity to the environment [72,73], whether positive or negative. This is in line with a diathesis–stress model, with highly sensitive children being more susceptible to both negative and positive environmental influences in relation to well-being [3].

### 4.1. Study Limitations

The present study has a number of limitations. First of all, the sample size is limited, and even though all children were individually interviewed, more variables could have been included with a larger sample. For example, measures of children’s state and trait anxiety as well as environmental and biological sensitivity could have been included [74]. These measures could add to the present findings by making interpretation easier and possibly better clarifying the role of the interaction between BMI and physiological response to a challenging stressful task in children’s well-being. However, it should be noted that given the children’s age, the above-mentioned constructs might be too difficult to self-report on and parents might have to be involved. Furthermore, children’s cognitive ability could have been included among studied variables. It is well known that cognitive functioning is related to well-being as well as to cardiac vagal activity [75], BMI [76] and classroom climate [77]. For example, cognitive flexibility (e.g., inhibitory control and shifting abilities) might interact with physiological regulation in promoting a healthy BMI and increasing children’s well-being. However, following Thayer’s neurovisceral integration model [78], we might expect a strong link between vagal activity and cognitive functioning; that is, by recording cardiac vagal activity we may have included a relevant correlate of children’s cognitive regulatory resources. Moreover, a bigger sample, involving more classrooms, would have allowed the assessment of classroom climate at a group level and use a nested design, thus better understanding the role of this variable as an actual measure of overall climate (as, for example, observed or reported by teachers). Furthermore, we here report exploratory analyses, which should be replicated in order to have more solid findings; and with a bigger sample size we could have split the sample in half randomly to check if the results were similar. In addition, a further limit is represented by the study cross-sectional design that does not allow causal inferences. Further study should include a larger sample size in order to have more generalizable findings (possibly with stronger effects), and longitudinal designs to study how the interaction among classroom climate, physiological self-regulation and BMI influences children’s development and well-being. Specifically, a longitudinal design with a larger sample size would allow testing changes in BMI in relation to both environmental and individual variables, giving the chance to understand more accurately the role and power of both classroom climate and self-regulation as well as their impact on body weight and well-being.

### 4.2. General Conclusions

Despite these limits, the study findings add to the literature underlining the role of social, psychophysiological and biological factors on children’s emotional and physical well-being. Most importantly, the moderating role of BMI as a continuous variable emerged. The present data highlight that normal weight variations, which are not necessarily related to underweightness or obesity, in interaction with vagal withdrawal as well as children’s perception of classroom climate, are interconnected with children’s well-being. Health professionals and policymakers should monitor children’s BMI even when it is not within a pathological range, as it could be related to how children respond to common potentially distressing events in their life (such as school oral tests), or how they adapt to specific environments (such as classroom climate), and more generally to their well-being.

## Figures and Tables

**Table 1:** Descriptive statistics and correlations and for all study variables (N = 142).

	2	3	4	5	6	7	*M* (*SD*)	Range
1. Physical Comfort	0.283 **	0.186 *	−0.066	−0.077	0.090	−0.031	3.91 (0.77)	1.2–5
2. Emotional Comfort		0.146	0.078	−0.219 **	−0.021	−0.065	3.86 (0.74)	2–5
3. Classroom Climate ^a^			0.084	−0.059	−0.214 *	0.169 *	1.44 (0.22)	0–1.61
4. rMSSD change				−0.043	−0.212 *	0.076	−7.93 (24.61)	−88.80–107.70
5. zBMI					0.151	−0.019	0.08 (0.92)	−2.10–3.81 ^d^
6. Age ^b^						−0.031	6.82 (0.71)	6–8
7. Gender ^c^							63 (44%) boys	

Note. ^a^ The variable has been log transformed to guarantee a good distribution; ^b^ Age is expressed in years; ^c^ 1 = male, 2 = female. rMSSD = square root of the mean squared differences of successive heart periods; ^d^ overweight from +1.04 to 1.64 and obese >1.64 obese. * *p* < 0.01; ** *p* < 0.001.

**Table 2:** Three best fitting linear regression models for variables predicting children’s physical and emotional comfort.

Panel A: Summary of Regression Analysis for Variables Predicting Physical Comfort			
Predictor	*B* (*SE*) ^a^	*p*	η^2^_p_
rMSSD change	−0.036 (0.017) *	0.033	0.002
Classroom Climate	0.694 (0.300) *	0.023	0.040
zBMI	−0.005 (0.023)	0.825	0.007
rMSSD change x zBMI	0.002 (0.009) *	0.038	0.033
Total R^2 a^	0.15		
N	138		
**Panel B: Summary of Regression Analysis for Variables Predicting Emotional Comfort**			
**Predictor**	***B*** **(*SE*)*^a^***	***p***	**η^2^_p_**
Gender ^b^	−0.237 (0.128)	0.066	0.026
rMSSD change	−0.036 (0.017) *	0.031	0.011
Classroom Climate	3.138 (1.230) *	0.012	0.029
zBMI	0.164 (0.100)	0.102	0.064
rMSSD change x zBMI	0.002 (0.009) *	0.018	0.043
Classroom Climate x zBMI	−0.155(0.071) *	0.032	0.036
Total R^2 a^	0.15		
N	139		

Note: ^a^ Unstandardized coefficient. ^b^ Gender coded 1 = male and 2 = female. * *p* < 0.01.

## Data Availability

The dataset generated during and/or analyzed during the current study is available from the corresponding author upon reasonable request.

## Supplementary Materials

The following are available online at https://www.mdpi.com/article/10.3390/ejihpe11040112/s1, Figure S1: BMI and BMI z scores frequency of distribution within the sample, Figure S2: Interaction Effect of rMSSD change and BMI on the Physical Comfort (N = 130), Figure S3: Interaction Effect of rMSSD change and BMI (Panel A) and of satisfaction with classroom climate and BMI (Panel B) on Emotional Comfort (N = 130).
