# Supplementary material for: Psychophysiological Regulation and Classroom Climate Influence First and Second Graders’ Well-Being: The Role of Body Mass Index
Source: Eur J Investig Health Psychol Educ. 2021 Dec 3;11(4):1581–98. doi: 10.3390/ejihpe11040112 (PMC8700215; doi:10.3390/ejihpe11040112)

Supplementary Figure S1

BMI and BMI z scores frequency of distribution within the sample.

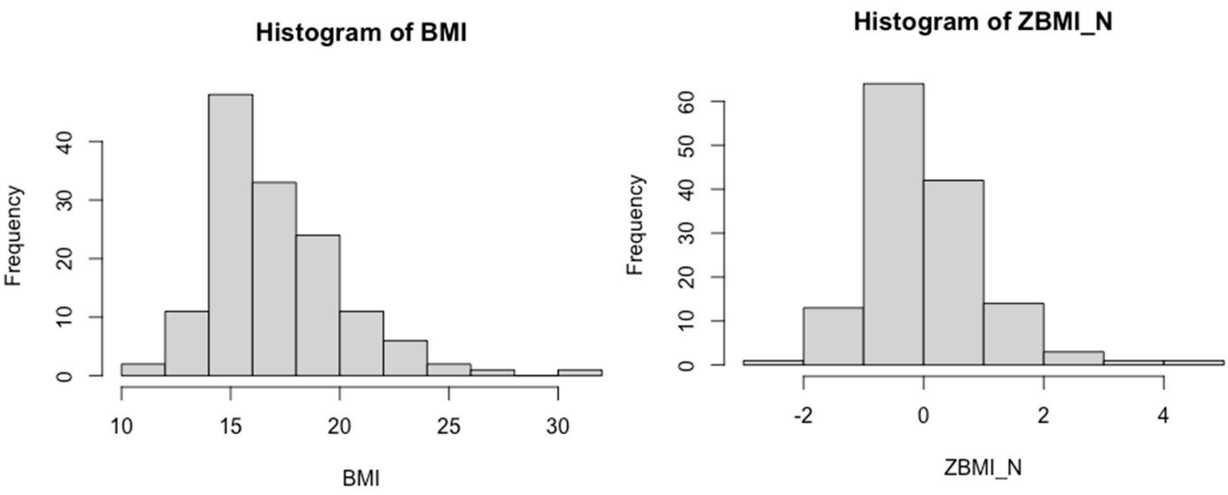

Supplementary Figure S2

Interaction Effect of rMSSD change and BMI on the Physical Comfort (N = 130).

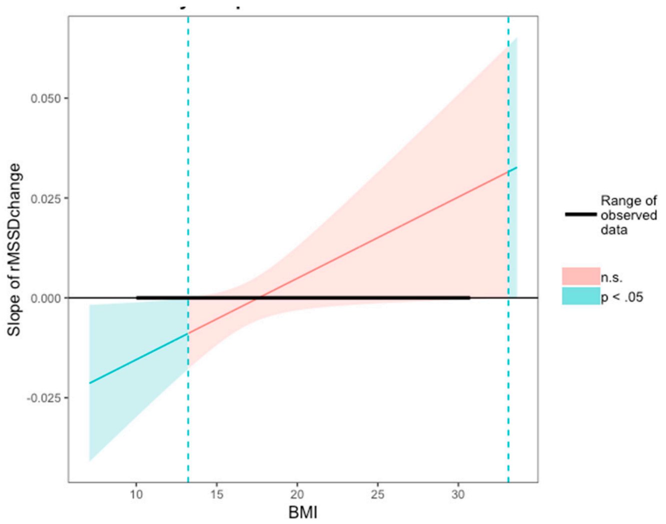

Supplementary Figure S3

Interaction Effect of rMSSD change and BMI (Panel A) and of satisfaction with classroom climate and BMI (Panel B) on Emotional Comfort (N = 130).

Panel A

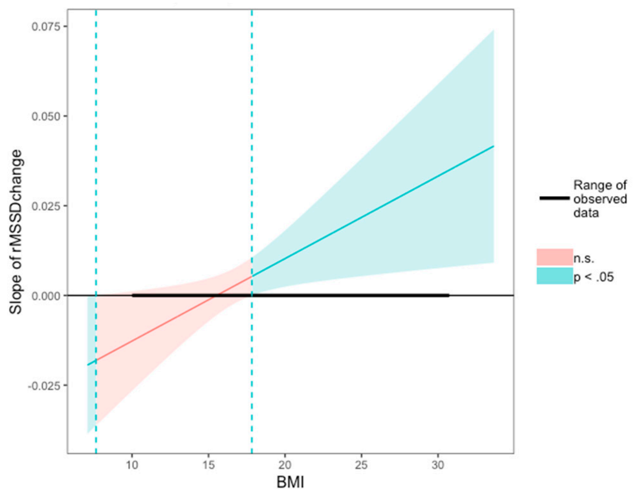

Panel B

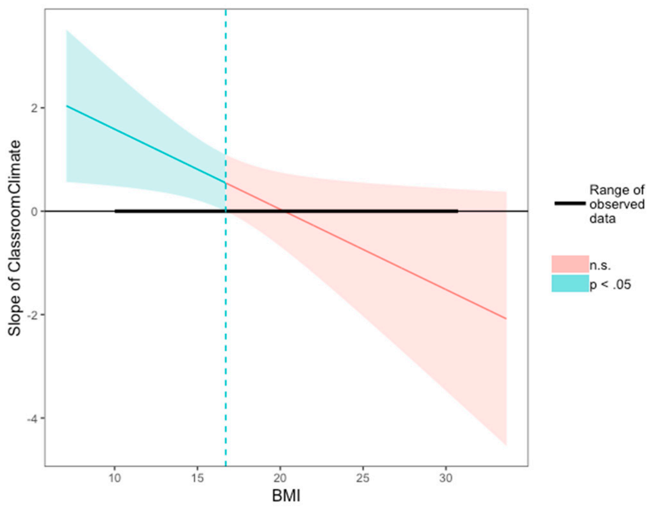

Supplement: Supplementary file 1 [file ejihpe-11-00112-s001.zip › ejihpe-1456879-supplementary.pdf]
